# *Dactylorhiza hatagirea* (D. Don) Soo: A Critically Endangered Perennial Orchid from the North-West Himalayas

**DOI:** 10.3390/plants9121644

**Published:** 2020-11-25

**Authors:** Ishfaq Ahmad Wani, Vijay Kumar, Susheel Verma, Arif Tasleem Jan, Irfan A. Rather

**Affiliations:** 1 Department of Botany, Baba Ghulam Shah Badshah University, Rajouri 185234, India; waniishfaq680@gmail.com; 2 Department of Biotechnology, Yeungnam University, Gyeongsan, Gyeongbuk 38541, Korea; vijaycbt@ynu.ac.kr; 3 Department of Biological Sciences, Faculty of Science, King Abdulaziz University (KAU), Jeddah 21589, Saudi Arabia; 4 Centre of Excellence in Bionanoscience Research, King Abdulaziz University (KAU), Jeddah 21589, Saudi Arabia

**Keywords:** antibiotic resistance, *Dactylorhiza hatagirea*, germplasm conservation, natural compounds, overexploitation

## Abstract

*Dactylorhiza hatagirea* (Orchidaceae) is a perennial herb inhabiting sub-alpine to alpine regions, ranging at elevations between 2500 and 5000 m.a.s.l. With palmately lobed rhizome and lanceolate leaves having a sheathing leaf base, it bears pink flowers with purple-colored notches and a curved spur. It finds wide use in ayurveda, siddha, unani, and folk medicine in curing disorders of the circulatory, respiratory, nervous, digestive, skeletal, and reproductive systems, besides boosting the immune system to fight infectious diseases. Secondary metabolites such as dactylorhins A–E, dactyloses A–B, and others exhibit a wide spectrum of pharmacological activities (antioxidant, antimicrobial, antiseptic, anticancer, and immune enhancing activities). Its use as a dietary supplement was found to be beneficial in increasing testosterone levels, resulting in improved sexual desire and arousal. Incessant overexploitation of this medicinally important herb has resulted in the dwindling of its populations in the wild, which has resulted in its classification as a critically endangered plant species. Efforts involving mass reproduction through in vitro (through tissue culture) and in vivo (by vegetative propagation) means are currently being made to maintain the germplasm of this critically endangered orchid. Holding immense significance in clinical research and drug discovery, work on the genomic front (transcriptomics) has recently been carried out to discover the wealth of unexplored genetic information for this perennial herb. The present study is aimed at reviewing different aspects of the orchid to present collective (summarized) information on this medicinally important herb in the present, particularly its botany, ethnobotanical uses, phytochemistry, and pharmacognosy, along with the strategies that need to be adopted to prevent its overexploitation in natural habitats.

## 1. Introduction

The Himalayas, extending into the Indian sub-continent, are considered to be a hotspot of biodiversity. Harboring 17,500 species of plants, modern and traditional medical practices (ayurveda, unani, and siddha) make use of almost 6000 of them [1,2]. They represent a remarkable contribution to the pharmaceutical field. Their trade in India is projected to be an estimated USD 1 billion per year [3,4]. Contributing indispensable raw material (flavonoids, alkaloids, saponins, etc.) for use in the formulation of different drugs, the demand for plants with ethnomedicinal importance has lately shown a surge, with wider employment in pharmaceutical practices. Aimed at maintaining individuals’ health, plant-derived substances (PDSs) such as taxanes, taxol, and cepholomannine from *Taxus brevifolia* Nutt. [5], diphyllin from *Diphylleia grayi* F. Schmidt [6], jatrophane from *Euphorbia semiperfoliata* Viv. [7], thymo-quinone and dithymoquinone from *Nigella sativa* l. [8] vinblastine from *Catharanthus roseus* (L.) G. Don, and others play a pivotal role in curing different diseases. The use of PDSs in disorders such as memory loss, osteoporosis, and age-related problems along with their function in boosting the immune system has broadened their potential in terms of use in modern healthcare systems [9].

As a preferable alternative to semi-synthetic drugs, natural products of plant origin are promoted as potential options for safer medicines and other life-saving drugs. The pharmaceutical use of medicinal plants with negligible side effects presents the possibility of their widespread application in mitigating the large fatalities associated with different deadly diseases [10]. Presently, plant species such as *Dactylorhiza hatagirea* provide an exemplary model for studying orchids as part of their ethnopharmacological properties and therapeutic applications. Owing to its immense significance in clinical research and drug discovery, the plant (tubers, leaves) has been used to study its potential to induce anti-inflammatory [11], anti-pyretic [12], anti-cancerous [13], neuropharmacological [14], and other effects. In the context of global health concerns, this medicinally important herb has broadly been used in the evaluation of its ethnopharmacological applications towards validating its efficacy and efficiency for use in combatting different diseases. The present study puts forth a summary of information on the botany, taxonomy, chemistry, and ethnopharmacology of *D. hatagirea*. As overexploitation of the plant for use in pharmaceutics has drastically reduced its populations and has brought it under threat, a section of the manuscript is dedicated to approaches adopted for its conservation and long-term survival in its natural habitat. 

### Methodology

The article covers the literature available from 1984 to 2020. The information was located, selected, and extracted from scientific journals, books, thesis, and reports via library and electronic search (PubMed and other search engines). Documentation of the available information from the literature helped in drafting different sections of the manuscript, such as morphology, taxonomy, and others, along with ethnopharmacological uses that depict its importance in the present day.

## 2. Orchids in Medicine: Special Reference to *D. hatagirea*

Orchidaceae is a family of angiosperms with around 25,000–35,000 species and 800 genera [15]. Orchids received their recognition through herbal writings from Japan and China [16]. The Chinese were the first to cultivate and describe their use in the healthcare system [17]. Orchids such as *Anoectochilus formosanus* Hayata from Taiwan [18], *Bletilla formosana* (Hayata) Schltr. from China [19], *Bulbophyllum kwangtungense* Schltr. from Japan [20], *Bulbophyllum odoratissimum* (J.E. Sm.) Lindl. from Thailand [21], *Calanthe discolour* Lindl. from Korea and Malaysia [22], *Catasetum barbatum* Lindl. from Guianas, Japan and Paraguay [23], *Coeloglossum viride* (L.) Hartm. from Tibet [24], *Cypripedium macranthos* Sw. from Mexico, Guatemala, and Colombia [25], *Dendrobrium* sp. from China, Japan, Taiwan, and Australia [26], *Listera ovata* (L.) R. Br. from Spain [27], *Maxillaria densa* Lindl. from Mexico [28], *Nidema boothii* (Lindl.) Schltr. from Malaysia [29], *Spiranthes australis* (R. Br.) Lindl. from Trinidad and Tobago, and *Vanda tessellate* (Roxb.) ex G. Don from India, Sri Lanka, and Burma [30] have made a significant impact, as their derivates (crude extracts as well as secondary metabolites) are used to treat various diseases.

Orchids are recognized throughout the world for the production of compounds used in the treatment of different diseases. In India, 1141 species belonging to 166 genera of orchids have been reported [31]. Here, the use of orchids in medicinal practices dates back to Vedic times. In addition, some members of Orchidaceae family are used to treat nerve disorders, fever, bone fractures, general weakness, tuberculosis, and other dermal problems [32,33]. *D. hatagirea* finds its use in a wide array of medicinal practices [34]. As per the published records, *D. hatagirea* is used for the treatment of amala pitta (gastritis), madhya bhangaasthi (bone fracture), jvara (fever), vajikarana (erectile dysfunction), haima (cold), bhishajyati (wound healing), and ayurdamah (nerve tonic). With the growing research in the biopharmaceutical and drug industry, extraction of the secondary metabolites and antioxidants from this plant has increased its demand [35].

## 3. Morphological and Anatomical Features of *D. hatagirea*

**Figure 1:**
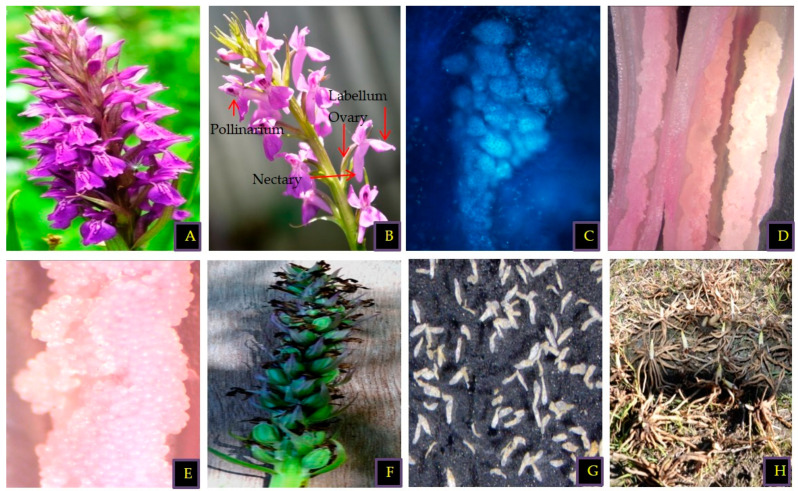
Progressive developmental stages of *D. hatagirea*: (**A**) inflorescence showing acropetal arrangement of flowers, (**B**) floral structure, (**C**) pollinarium bearing large number of pollen grain tetrads (captured on Nikon Eclipse 80 Fluorescence microscope at 10× magnification), (**D**) vertical section of trilocular ovary (captured on Nikon-C-FLED2 Stereo zoom at 10 × 1.7× magnification), (**E**) ovules (captured on Nikon-C-FLED2 Stereo zoom at 10 × 3.9× magnification), (**F**) fruit formation, (**G**) immature seeds (captured on Nikon-C-FLED2 Stereo zoom at 10 × 2.8× magnification), (**H**) tubers.

*D. hatagirea*, an important medicinal herb belonging to the Orchidaceae family, is commonly known as Himalayan marsh orchid [36]. It is a perennial herb confined to alpine regions at an elevation of 2500–5000 m.a.s.l. [37,38]. *D. hatagirea* bears slightly flattened, 3–7-fingered, palmately lobed, creamish-colored, tuberous roots measuring 5–12 ± 3.3 cm in length. The peduncle is generally 27–41 ± 6.7 cm tall. Leaves are acuminate or apex obtuse, linear-lanceolate to oblong clustered and sub-opposite towards base. Flowers grow in dense spike inflorescences and are zygomorphic, having fused male and female reproductive organs (a condition referred to as gynostemium, column, or gynostegium) (Figure 1). Flowers are pink, bearing purple-colored notches with six free perianth segments. The innermost segment forms an enlarged, pink-colored and sculptured labellum, which acts a resting pad for the pollinators, while the rest of the segments are similar in shape and form. Flowers show resupination at 180°, which brings the labellum perpendicular to the ovary. Flowers bear two dark green-colored pollinaria covered inside the anther cap. Each pollinarium bears a central sterile axis which combines with the pale yellow-colored caudicle (stalk). Caudicles at the upper side are separated by rostellum; however, at their base, they are joined by a sticky structure known as retinacula or viscidia. The ovary is tricarpellate, inferior, twisted and consists of one chamber with parietal placentation, having a large mass of ovules. Fruits are loculicidal capsules and minute seeds are generally liberated as immature embryos at the globular stage. 

## 4. Distribution, Trade, and Consumption

*D. hatagirea* is regarded as an Asian species of the genus *Dactylorhiza* [39]. It is distributed across India, China, Pakistan, Iran, Afghanistan, Tibet, Bhutan, Europe, North Africa, Temperate Asia, Mongolia, and Nepal [37,40,41]. In India, the plant is found in Jammu and Kashmir, including Ladakh, Uttarakhand, Himachal Pradesh, Arunachal Pradesh, and Sikkim [42,43,44,45,46]. Over the years, the market value of crude drugs obtained from the plant has shown an increasing trend which has led to the expansion of its market across different Indian states [13,47,48,49]. The huge demand in the pharmaceutical sector has driven a flourishing trade of around USD 71,583 [50]. Kala [51] reported that the annual demand for *D. hatagirea* is approximately 5000 tons, due its use in both traditional as well as modern medicine. Around ~7.38 tons of salep (processed tubers) obtained from *D. hatageria* are consumed annually to cure different ailments [41]. Overexploitation of the plant has drastically reduced its populations and has brought it under threat. Based on the number of existing populations and the area under its cover, Conservation Assessment and Management Plan (CAMP), Convention on International Trade in Endangered Species of wild flora and fauna (CITES), and International Union for Conservation of Nature (IUCN) have placed this plant under different categories of threat (Table 1).

## 5. Ethnopharmacological Importance of *D. hatagirea*

**Figure 2:**
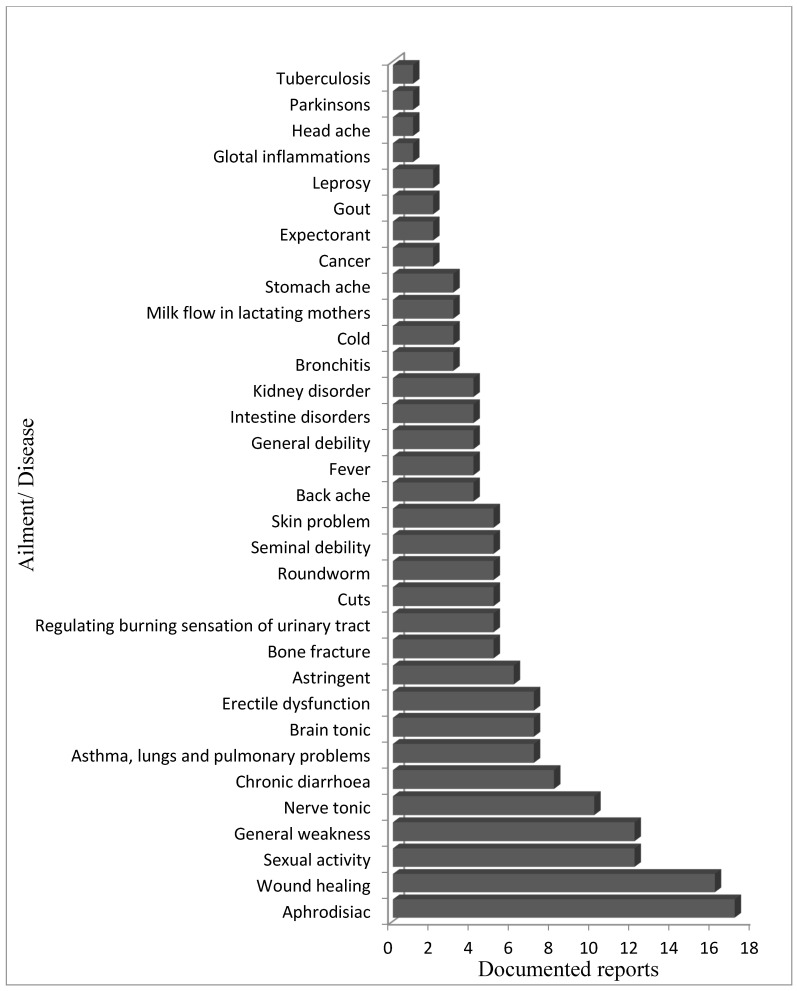
Use of *D. hatagirea* against different ailments as per available literature from 1964 to 2020.

Salep obtained from tubers (77%) and leaves (23%) is used in curing ailments like dysentery, chronic diarrhoea, etc. [57], besides being used as a nerve tonic, emollient, demulcent, astringent, and aphrodisiac [58]. It is also useful in treating general debility, emaciation, seminal weakness, neurasthenia, and cerebropathy [59] (Figure 2). A decoction of the tubers is helpful to relieve colic pain and fever, besides for speckling over cuts, burns, and wounds to stop bleeding. Testosterone levels were found increased in adult male rats after giving lyophilized extract of the plant, which increased their sexual behavior. [35,60]. Thakur and Dixit [35] reported that processed tubers of *D. hatagirea* significantly increase the functioning of sex organs by increasing the genesis of steroids (testosterone) hormones. Table 2 shows comprehensive information of the pharmacological uses of *D. hatagirea.*

## 6. Bioactive Compounds of *D. hatagirea*

Extract of tubers yields albumin, butanedioic acid, hydroquinone, lesoglossin, militarrin, pyranoside, pyrocatechol, and volatile oil. The antioxidant property of compounds obtained from the plant finds its use in the treatment of different human diseases. Indole alkaloids, phenolics (stilbene, e.g., resveratrol), and saponins along with ascorbic acid, phyllo- and naphthloquinones, glucomannan, and carotenoids [50] form the active constituents of *D. hatagirea*. Dactylorhins A-E (glycosidic compounds: dactylorhin A (C_40_H_56_O_22_)_,_ dactylorhin B (C_40_H_57_O_23_)_,_ dactylorhin C (C_14_H_24_O_10_)_,_ dactylorhin D (C_27_H_40_O_17_)_,_ dactylorhin E (C_27_H_40_O_17_)) and dactyloses A-B (glycosidic compounds: dactylose A, dactylose B, C_12_H_16_O_6_) obtained from *D. hatagirea* exhibit a wide range of pharmacological activities [80] (Table 3).

## 7. Pharmacological Importance of *D. hatagirea*

*D. hatagirea* is a high-value orchid with a broad range of phytochemicals which exert a wide range of beneficial effects. Information on the critical medicinal benefits of *D. hatagirea* use is listed below.

### 7.1. Antibacterial Activity

The root and shoot extracts are effective in treating a broad range of diseases caused by Gram-positive and Gram-negative bacteria [81]. Salep was found to be highly effective against *Escherichia coli* and *Shigella flexinerai,* which often show resistance to synthetic medicines [82]. Different extracts of *D. hatagirea* prepared with petrol, ether, chloroform, methanol, and water were tested against five different bacteria for the determination of zone of inhibition (ZOI) and minimum inhibitory concentration (MIC) [83]. Petroleum ether extract and chloroform extract of the aerial part and methanolic extract of the rhizome of *D. hatagirea* showed significant activity against *Escherichia coli.* The chloroform extract showed the best inhibitory action against *E. coli*, while these extracts show similar effects for *Shigella flexinerai*. The results indicate that the rhizome part is more effective than the aerial parts against all tested organisms. Besides this, *D. hatagirea* extract exhibits greater ZOI than ciprofloxacin against *S. aureus*. *D. hatagirea* exerts equal effect to that of norfloxacin for *S. aureus*. For *E. coli,* the ZOI of the aerial part of *D. hatagirea* was found to be almost equal to ciprofloxacin. For *S. flexinerai*, *P. aeruginosa,* and *B. subtilis,* the ZOI is equal for tuber extract and ciprofloxacin [81]. The antibacterial activity of root and shoot ethanol and methanolic extracts of *D. hatagirea* against *B. subtilis*, *S. aureus*, *E. coli*, and *P. aeruginosa* showed MIC at lower values [84]. Its ability to exert an effect that is comparable to the available regimes of medicines reflects its potential for use as effective antibacterial agent.

Bacterial drug resistance could be overshadowed by resistance provided by efflux pump inhibitors [85]. The efflux pumps are specific and possibly remove either a single class or several classes of antimicrobial compounds [86]. Pumps from the major facilitator superfamily (MFS) of Gram-positive bacteria play a key role in the efflux [87]. Plants being important sources of phytoconstituents raises the prospects of acting as a source of novel chemotherapeutic compounds, especially efflux pump inhibitors. Aqueous ethyl acetate and methanolic extracts of *D. hatagirea* exhibited efflux pump inhibitory activity against *S. aureus* strains. Efflux of EtBr and uptake of berberine results in a synergistic effect of each extract with ciprofloxacin and norfloxacin [88]. The use of plant extracts to inhibit multiple-resistance bacteria (MDR) [89] and prevent oral bacterial growth of *Cymbopogon* [90,91] and respiratory tract (RTIs) [92,93], urinary tract [94,95], cutaneous [96], and digestive infections [97] has been studied extensively. The results clearly show the applications of plant-based products as a substitute for antibiotics in overcoming various bacteria-related complications. The capability of a plant to exert a significant effect on efflux pumps expands the potential for its use as a potent efflux pump inhibitor. Besides this, its effectiveness against MDR bacterial isolates open avenues for its use in the design of drugs that can be employed to overcome the problem of drug resistance.

### 7.2. Anti-Inflamatory Activity

Alkaloids, flavonoids, glycosides, steroids, diterpenes, saponins, and tannins present in the tubers of *D. hatagirea* show potent anti-inflammatory activity. The activity was assessed in a rat paw oedema model induced by carrageenan and a cotton pellet granuloma model for acute/chronic inflammation. Adult Wistar rats of either sex responded better to hydroalcoholic extracts of *D. hatagirea* as compared to standard (aspirin 100 mg/kg and Indomethacin 10mg/kg). The extract showed significant anti-inflammatory effects in both acute and chronic inflammatory conditions [98]. Hydroethanolic extract of *D. hatagirea* tubers showed dose-dependent anti-inflammatory responses in the carrageenan-induced oedema among Wistar rats [11]. Their anti-inflammatory activity needs further exploration in terms of understanding the mode of operation for its utilization in the design of potent anti-inflammatory drugs for use in clinics.

### 7.3. Neuropharmacological Activity

Soporific drugs, commonly known as sleeping pills or hypnotic drugs, are a class of psychoactive drugs whose primary function is to induce sleep for the treatment of insomnia (sleeplessness) or as surgical anesthesia. The average percentage yield of hydroalcoholic extract of *D. hatagirea* revealed that its extract was safe at all doses when administered orally to mice, with no mortality. Besides this, the dose-dependency prolonged the duration of sleeping time among the tested animals compared to normal [14]. However, to gain in-depth insights into the mechanisms involved, further studies on animal models are needed before being employed as effective molecules in the treatment of neurological problems in humans.

### 7.4. Anti-Cancerous Activity

The extract obtained from *D. hatagirea* shows a considerable effect on cancerous cell lines. The population of Michigan Cancer Foundation-7 (MCF-7) and breast cancer (MDA-MB-231) cell lines grown in Dulbecco’s modified Eagle medium (DMEM) and 1% antibiotics with fetal bovine serum (FBS), respectively, showed a considerable decrease in their population [13]. However, human embryonic kidney (HEK-293) cell lines (normal cell line) grown in Leibovitz (L-15) medium show negligible effect. Root extracts of *D. hatagirea* show higher anti-cancerous potential than shoot extracts [13]. MDA-MB-231 cells treated with 1000 µg/mL tuber extract and shoot extract showed 82.38% and 83.81% viable cells, respectively [13]. Similarly, in the case of the MCF-7 cell line, 84.24% viability was found in the cells which were treated with 1000 µg/mL of tuber extract as compared to 87.09% of viable cells obtained from shoot extract treatment [13]. However, further study needs to be conducted using different animal models, under stress conditions, to understand its effects on different signaling pathways. Factors underlying the occurrence of the diseases should be explored in order to elucidate the mechanisms for devising effective treatment regimes.

### 7.5. Anti-Diabetic Activity

Antihyperglycemic agents extracted from the leaves and tubers of orchids make the plant ideal for compounds with anti-diabetic properties [99]. Anti-diabetic properties of *D. hatagirea* methanolic leaf extract using 3T3-L1 cell line showed no cytotoxic effect. The extract exhibited anti-diabetic properties, manifested by the inhibitory effect on α-amylase and α-glucosidase enzymes, enhancing the cellular uptake of glucose by inducing the expression of glucose transporter type 4 (GLUT4) on the cell surface. Inhibition of α-amylase activity occurs at 31.25 µg/mL and 74.53 ± 0.5% inhibition at 500 µg/mL concentration with respect to the standard drug acarbose, which shows inhibition between 31.25 µg/mL and 85.27 ± 1.2%. Inhibition of α-glucosidase activity occurs between 30.16 ± 0.16% and 72.13 ± 0.78% with respect to standard drug acarbose, the positive control drug, which showed inhibition of 43.20 ± 0.09% and 94.41 ± 0.49% at 500 µg/mL. An analysis of the expression of GLUT4 using anti-mouse Glut4-FITC antibody (#NBPI-49533F, Novus Biologicals) with metformin (control) revealed that *D. hatagirea* extract-treated cells showed elevated levels of GLUT4 as compared to untreated cells [100]. Root extract shows a dose-dependent decrease in the blood sugar, total cholesterol, and total triglycerides and an increase in the total protein content [101].

Solvent-based extraction (chloroform, methanol, water, petroleum ether, ethanol) and photochemical screening of the secondary metabolites from *D. hatagirea* reveal antimicrobial and other lifesaving pharmaceutical applications. Effects of azithromycin and amikacin-resistant bacteria (*E. coli*) are highly neutralized by root and shoot extracts of *D. hatagirea* [81,84]. Root extracts show significantly greater potential than glibenclamide against increased levels of certain blood ingredients in Wistar rats [101]. Detailed applications of different extracts of *D. hatagirea* are shown in Table 4.

### 7.6. Other Applications

The tubers of *D. hatagirea* show wide utilization in silk industries for sizing material [102]. The plants are grown in gardens for decorative purposes. The aesthetically appealing appearance of the flowers makes them suitable for ornamental purposes (placed in flower vases, twisted within hair ponies, making bracelets and necklaces). Grounded stem and leaves are used as insect repellant. Leaves and stem of the plant are used as fodder for livestock. *D. hatagirea* helps in improving the flavor and taste, color and appearance, body and texture, and melting quality of frozen milk products. Moreover, young leaves and shoots are also used as vegetables [103]. Extract of the flowers is used in perfume industries to increase fragrance. Tubers of the plants are used for witchcraft [77].

## 8. Conservation Approaches

The medicinal property of *D. hatagirea* plant has led to exploitation of its populations in nature [104]. Owing to the dwindling populations and inability of the seeds to germinate without mycorrhizal association [105], its efficient conservation is a challenging task to accomplish. However, the successful introduction of the plant by symbiotic seed germination under in vitro conditions was achieved by Aggarwal and Zettler [106]. Novotna et al. [107] established seedling development of *D. hatagirea* using 10 g dm-3 sucrose and glucose treatment. Further, treatment with N6-benzyladenine or cytokinins N6-(2-isopentenyl) adenine and their amalgamation amid auxin (IBA) helped in significant development of shoots. Several researchers carried out in vitro culturing of immature seeds for large-scale production of *D. hatagirea* [13,105]. The protocorms so formed were then cultured on rejuvenation medium with MS and BM-2 media enriched with various plant growth hormones (KN-3mg/l IBA-3 mg). For adaptation, seedlings were transferred to a blend of cocopeat, perlite, and vermiculite in the ratio of 1:1:1. The saplings formed were finally moved to a greenhouse. Plant multiplication and conservation of the plant by spliced tuber plantation were performed by Shrestha and Shrestha [108]. Green pod culture of *D. hatagirea* was performed by Giri and Tamta [109] in different growing media such as Vejsadova (VJ), Vacin and Went (VW), Murashige and Skoog (MS), and Knudson C (KC). Better results for seed germination were obtained in MS medium enriched with activated charcoal, morpholino ethane sulphonic acid, and peptone. Due to the threat of extinction, *D. hatagirea* is protected in different countries, including Luxembourg, Belgium, the United Kingdom, and Nepal [110]. As per the Forest Act 1993, Forest Regulation Act 1995, and its amendment in 2005, the collection and trade of tubers of *D. hatagirea* is banned in Nepal. However, collection must follow the regulations for the trade of *D. hatagirea* as per Forest Regulation 1995 and its amendment in 2005 [13].

## 9. Conclusions and Future Perspectives

**Figure 3:**
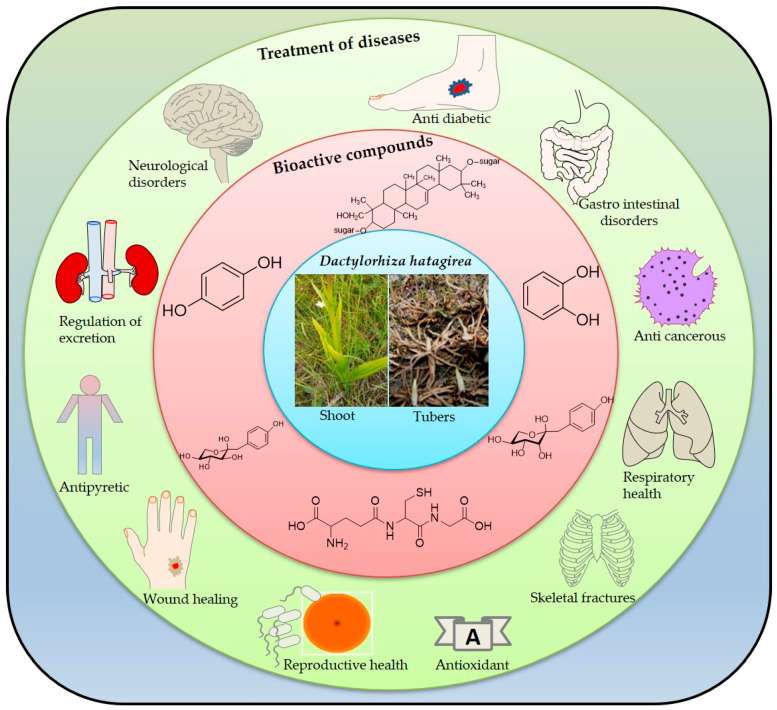
Representation of bioactive compound-mediated pharmacological effects of *D. hatagirea* in humans. Note: The figure simply depicts the compounds obtained from *D. hatagirea* and the effects observed, without correlating them to each other in the figure.

The ethnomedicinal properties of the plant reveal its widespread utilization in traditional medicine systems. The use of plants to cure ailments such as chronic diarrhea, fractured bones, seminal debility, erectile dysfunction, gout, Parkinson’s disease, tuberculosis, and stomachache is worth mentioning (Figure 3). Pharmacological studies on the secondary metabolites have confirmed its antibacterial, anti-cancerous, and testosterone-increasing potential. In the healthcare system, *D. hatagirea* acts as adjuvant therapy for the treatment of diseases and in maintaining good health. The current research should aim at analyzing the active constituents of the plant for their therapeutic potential by strengthening animal studies and performing clinical trials that could help in the subsequent formulation of the plant for use in modern medicine.

Further studies are needed for exploring the potential of the plant for unknown bioactive constituents, studying the underlying mechanisms of bioactive components, and in performing analyses of their efficacy and possible use in assisting the exploration of new therapeutic molecules. Additionally, new possibilities also need to be explored for using the natural constituents of the plant in complementing standard medicines as a possible solution to diminish the occurrence of diseases and in combatting the infections caused by multidrug-resistant microorganisms. The natural remedies of *D. hatagirea* and its essential ingredients may play a vital role in the development of novel drugs for the treatment of different human diseases. There is no doubt that the extraction of secondary metabolites and their use in the drug industry is gaining pace; however, more research is needed on this plant to validate its effectiveness in humans. In addition, studies on frontier disciplines of modern aspects of biology such as genomics (transcriptomics, metabolomics, etc.) are needed in order to gain a detailed insight into this medicinally important plant. Considering the vulnerability of this orchid, effective measures through mass multiplication under in vitro and in vivo conditions should be carried out to maintain the germplasm of this critically endangered orchid above its threshold level. Additionally, the areas with natural populations of *D. hatagirea* should be recommended as medicinal plant conservation areas (MPCA).

## Figures and Tables

**Table 1:** Status of *D. hatagirea* as for different categories of threat.

Status	Category	Reference(s)
Endangered	CAMP and CITES	[52]
Critically Endangered	CAMP and CITES	[53,54]
Vulnerable	CAMP	[53]
Listed among Appendix II	CITES	[55]
Critically rare	IUCN	[53,55]
Threatened	IUCN	[53]
NPSMHCC	MFSC, Kathmandu	[56]

**Abbreviations:** CAMP: Conservation Assessment and Management Plan, CITES: Convention on International Trade in Endangered Species of wild flora and fauna, IUCN: International Union for Conservation of Nature, NPSMHCC: National Priority Species of Medicinal herbs for Cultivation and Conservation, MFSC: Ministry of Forest and Soil Conservation.

**Table 2:** Traditional uses, area, and mode of application of *D. hatagirea*.

S. No	Ailment/Use	Plant Part	Place/Country	Mode of Application	References
1	Respiratory (asthma, bronchitis, lungs, and other pulmonary problems)	leaves and tubers	India (Ladakh, Gharwal Himalaya) Nepal (Dolpa, Rasuwa, Humla, Jumla, and Mustang districts)	- Decoction obtained from the tubers is mixed with boiled water and taken - Inhalation of stream of plant parts boiled in water - Dried tubers are mixed with other medicinal plants and boiled in water for daily consumption	[35,61,62,63,64,65,66]
2	Neurological (brain tonic, nerve tonic)	leaves and tubers	India (Gharwal Himalaya), Nepal	- Extract obtained from tubers and leaves is taken in the morning and after dinner - Decoction of the plant is consumed as juice	[35,63,65,67]
3	Digestive (stomachache, chronic diarrhea, intestinal disorders)	tubers	India (Gharwal Himalaya, Arunachal Pradesh), Nepal (Dolpa, Rasuwa, Humla, Jumla, and Mustang districts)	- Plant parts are boiled in water and the extract (crude drug) is used - Tubers of the plant are ground to fine powder, mixed with other medicinal herbs, and taken with milk or water.	[35,37,58,65,67,68]
4	Urinary (kidney disorders, burning sensation, and urine discharge)	tubers	India (Gharwal Himalaya)	- Unspecified	[53,61,69]
5	Sexual (sexual activity, seminal debility, erectile dysfunction)	tubers	India (Gharwal Himalaya), Pakistan (Gilgit), Nepal (Dolpa, Rasuwa, Humla, Jumla, and Mustang districts)	- Extract of the root is taken on empty stomach and after dinner to increase sexual activity	[70,71]
6	External uses (headache, wound healing, skin problems)	tubers	India (Gharwal Himalaya, Kuman Himalayas, Arunachal Pradesh), Nepal (Dolpa, Rasuwa, Humla, Jumla, and Mustang districts)	- Plant parts are crushed, mixed with turmeric, and applied externally - Powdered roots are spread over wounds to control bleeding - Tubers are ground into fine powder, mixed with mustard oil, and applied on wounds.	[65,72,73,74,75]
7	Others (backache, bone fracture, fever, weakness, general debility, milk flow in lactating mothers)	tubers and leaves	India (Gharwal Himalaya, Western Himalaya, Manali), Pakistan (Gilgit and Bugrot valley), Nepal (Rasuwa district)	- Tubers are powdered and mixed with mustard oil for use externally - Plant parts are boiled in water and their extract is dissolved in water and taken after meals.	[13,54,57,58,62,65,66,76,77,78,79,80]

**Table 3:** Structural aspects of secondary metabolites extracted from *D. hatagirea*.

Name	Synonym	Structure	References
1-deoxy-1-4 hydroxyphenyl-L-sorbose	Dactylose A	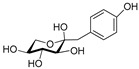	[80]
1-deoxy-1-4 hydroxyphenyl-L-tagatose	Dactylose B	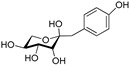	[80]
(2R)-2-β-D-glucopyranosyloxy-2(2-methylpropyl) butanedioic acid bis (4- β-D-glucopuranosyloxybenzyl) ester	Dactylorhin A	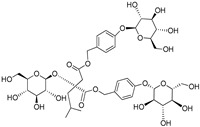	[80]
(2R-3S)-2- β-D-glucopyranosyloxy-3-hydroxy-2(2-methylpropyl) butanedioic acid bis (4 β-D—glucopyranosyloxybenzyl) ester	Dactylorhin B	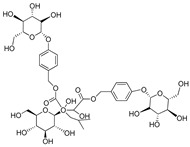	[80]
(2R)-2-β-D-glucopyranosyloxy-2(2-methylpropyl) butanedioic acid	Dactylorhin C	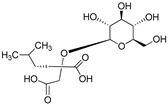	[80]
(2R-3S)-2- β-D-glucopyranosyloxy-3-hydroxy-2(2-methylpropyl) butanedioic acid 1-(4- β-D- glucopyranosyloxybenzyl) ester	Dactylorhin D	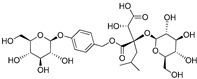	[80]
(2R)-2-β-D-glucopyranosyloxy-2(2-methylpropyl) butanedioic acid 1-(4- β-D-glucopuranosyloxybenzyl) ester	Dactylorhin E	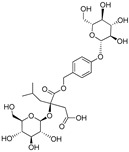	[80]
(E)-5-(4-hydroxystyryl) benzene-1,3-diol	Resveratrol	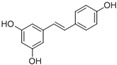	[50]
1H- Indole	Indole alkaloids	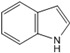	[50]
Napthalane -1,4- dione	Napthoquinone	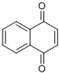	[50]
(R)- 5- ((S)- 1,2- dihydroxyethyl)- 3,4 dihydroxy furan-2 (5H)-one	Ascorbic acid	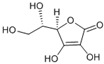	[50]
2- methyl- 3 ((7R, 11R,E), 3,7,11,15- tetra methylhexadec 2- en-1-yl) naphthalene-1,4-dione	Phylloquinone	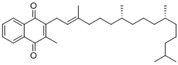	[50]
	Militarrin	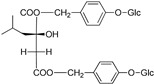	[80]
	Lesoglossin	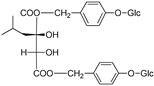	[80]
	Pyrocatechol	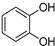	[80]
	Glucomannan	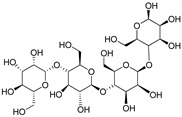	[50]
	Saponin	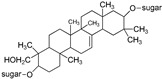	[50]

**Table 4:** Different activities observed for root and shoot-derived extracts of *Dactylorhiza hatagirea*.

Plant Extract/Antibiotics	Resistance Against	Dosage	Effect	Reference
**Antibacterial**				
Shoot extract	SA, EC, SF, PA, BS	500 mg/mL	Best inhibition for EC, better for SA, and good for SF, SA, PA, and BS	[81]
Root extract		500 mg/mL	Best inhibition for SF, better for SA, EC, BS, and good for PA	[81,84]
**Antioxidant**				
Root extract		3 μg/mL	Best antioxidant activity	[84]
Plant extract	FRAP	3%	Antioxidant activity	[102]
Root extract	NO, H_2_O_2_	NA	Better antioxidant activity	[103]
**Anti-inflammatory**				
Root extract	Carrageenan-induced paw oedema	100, 200, 300 mg/kg	Shows decrease in the volume of paw with increase in dosage	[11,98]
Root extract	Cotton pellet granuloma	100, 200, 300 mg/kg	Reduced granuloma formation with increase in dosage	[98]
**Neuropharmacological**				
Root extract	Hypnosis	100, 200, 300 mg/kg	Shows prolonged hypnosis with increase in dosage	[14]
**Anti-pyretic**				
Root extract	Brewer’s yeast induced pyrexia	100, 200, 300 mg/kg	The influence of pathogenic fever was decreased with dose-dependent concentrations	[12]
**Anti-diabetic Blood biochemical parameters and** **3T3-L1 diabetic cell line**				
Root extract	Blood glucose	100, 200 mg/kg	Shows dose-dependent decrease in blood glucose with increase in time	[101]
Root extract	TC, TG and TP	100, 200 mg/kg	TC and TG show dose-dependent decrease while TP shows increase with increase in dosage concentration	
Leaf extract	α amylase activity	31.25 and 500 μg/mL	α amylase activity decreased with increased dosage	[100]
Leaf extract	3T3-L1 diabetic cell line	25–400 μg/mL	3T3-L1 cell line viability decreased with increase in dosage	
Leaf extract	GLUT 4 expression and NBDG uptake	100 μg/mL	Increased expression	
**Anti-cancerous potential**				
Shoot extract	HEK- 239, MDA, MB- 231, MCF 7 cell lines	250–1000 μg/mL	Cell viability decreases with increased dosage	[13]
Root extract		250–1000 μg/mL	Cell viability decreases with increased dosage	

**Abbreviations:** SA, *Staphylococcus aureus*; EC, *Escherichia coli*; SF, *Shigella flexneria*; PA, *Pseudomonas aeruginosa*; BS, *Bacillus subtilis*; TC, total cholesterol; TG, total triglycerides; TP, total proteins; GLUT4, glucose transporter type 4; NBDG, 2-(N-(7-nitroben-2-oxa-1,3-diazol-4-yl)amino)-2-deoxyglucose; HEK, human embryonic kidney; MDA-MD, breast cancer cell line; MCF-7, Michigan Cancer Foundation; FRAP, ferric reducing antioxidant power; NO, nitric oxide; H_2_O_2_, hydrogen peroxide.
